# Distribution and patterning of non-communicable disease risk factors in indigenous Mbororo and non-autochthonous populations in Cameroon: cross sectional study

**DOI:** 10.1186/s12889-016-3837-8

**Published:** 2016-11-24

**Authors:** Nyuyki Clement Kufe, George Ngufor, George Mbeh, Jean Claude Mbanya

**Affiliations:** 1 South African Medical Research Council/University of the Witwatersrand Developmental Pathways for Health Research Unit, Department of Paediatrics and Child Health, Faculty of Health Sciences, University of the Witwatersrand, Johannesburg, South Africa; 2 Health of Populations in Transition (HoPiT) Research Group, Department of Medicine and Specialities, Faculty of Medicine and Biomedical Sciences, The University of Yaoundé 1, Yaoundé, Cameroon

**Keywords:** Indigenous populations, Fulani, Non-communicable diseases, Risk factors, Low and middle income countries

## Abstract

**Background:**

Data on Non-Communicable Diseases (NCDs) among indigenous populations are needed for interventions to improve health care. We conducted a survey in 2013 among rural indigenous Mbororo, Fulbe and other ethnic groups to determine the distribution of risk factors of NCDs in Cameroon.

**Methods:**

We selected seven targets of NCD risk factors: tobacco use, alcohol use, diet (salt/sugar intake, vegetable/fruit consumption), raised blood pressure, raised blood glucose, physical inactivity and weight measures. The WHO STEPwise approach was used to collect data from 1921 consenting participants aged ≥20 years. Prevalence of NCD risk factors was summarised by descriptive statistics.

**Results:**

Underweight was widespread, Mbororo (50.8%) and Fulbe (37.2%). Increase in prevalence of six risk factors was observed among the Fulbe when compared to Mbororo. Participants aged 20–39 years had low levels of physical activity, poor diet and higher levels of alcohol consumption (except Mbororo) and those aged ≥40 years had higher prevalence of diabetes, hypertension, current smoking and overweight/obesity. Men and women differed in current smoking, occasional/daily alcohol consumption, pre-hypertension and hypertension, continuous walking for at least ten minutes, and weight measures for Fulbe and Mbororo, *p* < 0.05.

**Conclusion:**

Distribution of NCD risk factors was high among settled Fulani (Fulbe) when compared to indigenous nomadic Fulani (Mbororo). Change from nomadic to settled life might be accompanied by higher prevalence of NCDs. This data should be used to develop intervention programmes to curb the rising burden of NCDs in rural indigenous and non-indigenous populations.

## Background

Non Communicable Diseases (NCDs) related deaths estimated at 35 million worldwide every year cause a large burden on individuals, families and health care systems [1, 2]. Four clusters of NCDs cardiovascular diseases (CVDs), cancers, chronic pulmonary diseases and diabetes) account for 80% of preventable deaths and disability. In Low and Middle Income Countries (LMIC) about 80% of premature deaths are related to NCDs and 90% of NCD related deaths occur before the age of 60 years, all age groups vulnerable [3]. The United Nations (UN) urged all member states to commit to prevention and control of NCDs [4]. Leading risk factors of NCDs include raised blood pressure, tobacco use, harmful alcohol consumption, physical inactivity, raised total cholesterol, raised blood glucose, overweight/obesity and low fruit/vegetable consumption accounting for about 80% deaths from heart disease and stroke [5].

Racial and ethnic disparities in health care, prevalence and risk factors of diseases are a complex problem in public health affecting mostly minority groups, the disadvantaged and indigenous groups. These disparities are partly due to bias, stereotyping, mistrust, socioeconomic differences, and health seeking attitudes, social and environmental determinants of health [6].

Indigenous populations are socially disadvantaged experiencing high rates of abject poverty, high unemployment, low education, overcrowded households, poor diet (unavailability of fruits/vegetables), higher rates of infectious disease burden especially amongst children and higher burden of life-style related NCDs amongst adults [7] and are experiencing transition from traditional to transitional and modern lifestyles witnessing increase prevalence of NCDs [7].

The United Nations (UN) Declaration on the Rights of Indigenous Peoples [8] and UN Secretariat of the Permanent Forum on Indigenous Issues [9] advocated to governments and World Health Organisation (WHO) to tackle diabetes and NCDs with an action plan focusing on prevention and access to care [10]. National minority groups recognised as indigenous people by the UN in Cameroon are Mbororo, Pigmies and Kirdi communities [10]. Both nomadic pastoral Fulani (Mbororo) and the settled Fulani (Fulbe) share a common language (Fulfulde) and ancestry and generally referred to as Fulani or Peul. The Peul are culturally diverse and the mostly widely dispersed people in Africa on the Sahel and Savannah parts of West and East Africa in Niger, Nigeria, Chad and Cameroon [11]. Social disadvantage observed among Fulani is associated with NCDs [12–17] especially poverty viewed by the Mbororo as lack of cattle and land [9], exclusion from community social life, being considered as less than equal or discriminated against results in worse health and higher risk of premature death [18].

Most populations are ethnically heterogeneous containing disparate subgroups and incidence of diseases, prevalence and complications vary from one ethnic group to another [19, 20]. Ethnic variations in disease burden are observed in the differential rates, individual responses to environmental conditions and risk factor profiles [21]. There is a dearth of knowledge on the distribution of risk factors of NCDs among indigenous populations of Africa. We analysed data from our 2013 survey on the distribution of seven risk factors of NCDs among Fulbe, Mbororo and the general population regrouping all other ethnic groups of Bantu ancestry in the same geographic area.

## Methods

### Setting and study population

This study was conducted under the auspices of the project *“Improve Access to Diabetes Care in Indigenous Fulani (Mbororo/Fulbe) populations of Adamawa and East Regions of Cameroon*” in the localities of Mandjou II, Guiwa Yangmou, Gom-Mana, Mazidou and Sabga.

### Sample size estimation

Sample size was calculated with an estimated prevalence of risk factors for this population of 0.50, precision of 0.05 and confidence level of 95%. We adjusted for design effect of 1.5, 20-year age groups for each sex, with additional 60+ age interval and considered two ethnic groups, thus set the number of new subgroups at three gave 1 728 participants and further adjusted for a 10% non-response rate [22], final sample size was 1 920.

### Sampling design

A multistage cluster sampling method was used. Five sites/localities inhabited mostly by Peul (Fulani) populations were selected based on accessibility by road in the East and Adamawa regions of Cameroon. The five localities were considered as five strata sampled by probability proportional to size (PPS) and the households as clusters from which a purposeful sample was drawn. A list was constituted from the census and all adults aged 20 years and above were invited to participate in the study through the household head and after individual informed consent. Further, each sampled participant was selected with equal probability from the house hold. In order to assure appropriate sample size in each site, each household was visited twice and if nobody accepted to participate a similar household was identified in the same site to the exclusion of the selected ones. All participants except pregnant women, mentally or physically challenged people and participants on diabetes medication ≥20 years having resided in the site for at least a year were invited for the study. Our census recorded 2702 participants, 2115 invited and 1921 participants took part in the study; response rate 90.8%, Fulbe 87.5%, Mbororo 85% and general population 92%.

### Data collection

#### Procedure

Participants were informed and asked to fast as from 22.00 h for the Fasting Capillary Glucose (FCG) test a day before. According to written standardised procedures, teams visited households from six in the morning to six in the evening and administered structured pre-tested questionnaires in French/English or local language and performed measurements. A household questionnaire administered to the household head or a senior member requested information on number of people in the household, wealth (land, cattle and income), common diseases and mortality. A WHO STEPwise approach to surveillance of NCDs (WHO STEPS, 2005) standardised instrument was used for data collection on socio-demographics, site, ethnic group, self-reported behavioural risk factors of NCDs, family history of diabetes, hypertension, and obesity, remedy taken if participant was diabetic, hypertensive or has tuberculosis, and blood pressure, FCG and anthropometric values taken.

#### Anthropometric measurements

Weight, height, waist and hip circumference were measured with participants in light clothing, without shoes and motionless according to standard methods described previously [23]. Standing height was measured with a wooden stadiometer to the nearest 1 mm and weight with a calibrated SECA scale to accuracy of 0.1 kg. Waist and hip circumferences were measured with inelastic fibreglass meter band to the nearest 0.1 cm.

Body Mass Index (BMI), gender specific Waist-Hip Ratio (WHR), and Waist Circumference (WC) were assessed and classified according to WHO guidelines [24–26].

#### Blood pressure measurement and definition

Blood pressure was measured on seated participant thrice on the right arm at five minutes interval; with uncrossed legs using arm blood pressure fully automated calibrated Omron M3 machine. Mean blood pressure of two closest measures was obtained. Participants with mean Systolic Blood Pressure (SBP) ≥ 140 mmHg and/or mean Diastolic Blood Pressure (DBP) ≥ 90 mmHg and/or self-reported treatment of hypertension with anti-hypertensive medication within last two weeks were considered hypertensive and classified according to guidelines [27, 28].

#### Blood glucose methods

Two measurements using standard protocol on different days of FCG were performed between six and ten in the morning with HemoCue Hb 201 DM Analyser (Angelhom, Sweden) for participants who had no caloric intake for at least eight hours [29]. International Diabetes Federation (IDF)/WHO diagnostic and classification criteria was used for two concordant results [30]. Participants with discordant results (less than 1%) were not included in the analysis.

Physical activity was assessed with the Global Physical Activity instrument included in the questionnaire. It measured low, moderate and vigorous physical activity based on the intensity, duration and frequency of physical activity in occupational and leisure times [31, 32].

Alcohol consumption was classified into abstainers (never consumed) and occasional (drank in the past 12 months) or daily drinkers.

Tobacco consumption considered manufactured or hand-rolled cigarettes, cigars, smoked, chewed or inhaled and classified participants into abstainers, current and daily smokers [33].

Fruit and vegetable intake was based on the frequency of intake per week. From zero to two times of intake/week it was classified as low, three to four times/week as moderate and five to seven times/week as high [34].

#### Data management and statistical analysis

Data was entered into Epi Data 3.0 platform and analysed using STATA 13 SE (StataCorp.2012. College Station, TX: StataCorp LP) taking into consideration the cluster sampling by use of survey command and stratification. We excluded data from partially completed questionnaires or participants whose FCG was not measured twice and/or had missing variables. Means (x̄) and standard deviation (±SD) were calculated. Chi-squared and Students t-tests performed with statistical significance of *p* < 0.05. One-way Analysis of Variance (ANOVA) was carried out testing the null hypothesis “the mean value of the outcome is the same across all the ethnic groups sampled from, against the alternative that the mean differ in at least two of the ethnic groups”. ANOVA Bonferroni’s post hoc tests were carried out to examine any statistical significant pairwise differences in means of variables between the groups. Direct age standardization using WHO New World Population as reference was done [35].

#### Role of the funding partner

The sponsor of the project did not play any role in the study design, protocol, data collection, analysis, interpretation, writing of this paper, decision to submit for publication or any aspect pertinent to this study.

## Results

### Descriptive results

**Fig. 1:**
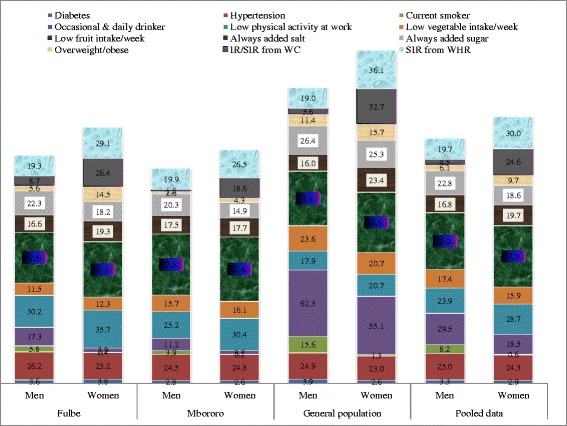
Percentage Cumulative Distribution and Patterning of NCD Risk Factors by Gender, 2013

**Fig. 2:**
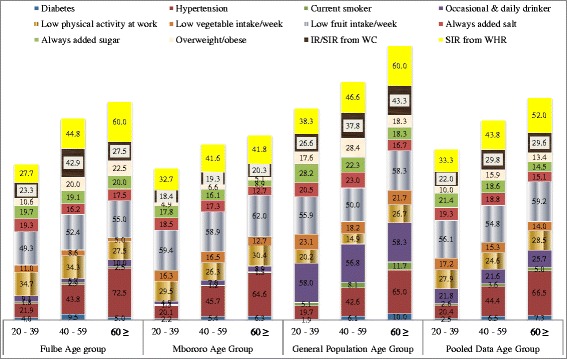
Percentage Distribution and Patterning of NCD Risk Factors by Age Group, 2013

A total of 814 households and 1921 participants aged ≥20 years were included in data analysis. Mean age of participants was 36.1 ± 14.4 (CI:35.4-36.7) years. The proportion of females was higher and the age group 20–29 years most represented. Questionnaires were often administered in the local Fulfulde language to Fulani. Mbororo households were the most crowded and the Mbororo were the recent arrivals at present site. About three quarters of Mbororo had annual income of less than USD 200 as compared to about two thirds of Fulbe and general population (Table 1). The Mbororo were the least educated (17.8%). Socio-demographic, health, lifestyle and family history characteristics by ethnic group are shown in Table 2. Table 3 shows variables with statistical significant differences between ethnic groups after post hoc test. Table 4 shows age and sex standardized prevalence of cardio metabolic risk factors by ethnicity and gender. Figure 1 shows cumulative percentage distribution of NCD risk factors and Fig. 2 percentage distribution by age interval in Fulbe, Mbororo, general population and pooled data.

**Table 1:** House hold data

	Fulbe, n (%)	Mbororo, n (%)	General population, n (%)	Pooled, n (%)	*p*-value
Number of households	182 (22.4)	373 (45.8)	259 (31.8)	814 (100.0)	
Number of people in household					
1–5	113 (62.1)	213 (57.1)	165 (63.7)	491 (60.3)	0.25
6–10	53 (29.1)	105 (28.2)	64 (24.7)	222 (27.3)	
10>	16 (8.8)	55 (14.7)	30 (11.6)	101 (12.4)	
Mean number of adults (20 years≥) in household (Min, Max^a^)	5 (1,27)	6 (1,35)	5 (1,24)	6 (1,35)	0.07
Mean number of years spent at present site (Min, Max)	10.1 (1,45)	9.9 (1,50)	11.3 (1,50)	10.4 (1,50)	<0.001
Number of years at site					
1–10 years	128 (70.4)	260 (69.7)	150 (57.9)	538 (76.0)	0.08
10 > years	54 (29.6)	113 (30.3)	109 (42.1)	276 (33.9)	
Cattle					
Non	162 (89.0)	320 (85.8)	248 (95.7)	730 (89.7)	<0.001
1 to 9 herds	4 (2.2)	18 (4.8)	8 (3.1)	30 (3.7)	
+ 10 herds	16 (8.8)	35 (9.4)	3 (1.2)	54 (6.6)	
Sheep/Goats/others					
Non	167 (91.8)	345 (92.5)	247 (95.4)	759 (93.2)	0.59
1 to 9 herds	10 (5.5)	19 (5.1)	8 (3.1)	37 (4.6)	
+ 10 herds	5 (2.7)	9 (2.4)	4 (1.5)	18 (2.2)	
Annual Income in US Dollars					
0 to199	117 (64.3)	277 (74.3)	161 (62.2)	555 (68.2)	<0.001
From 200 to less than 1 999	54 (29.7)	78 (20.9)	68 (26.2)	200 (24.6)	
From 2 000 to less than 5 999	9 (4.9)	13 (3.5)	23 (8.9)	45 (5.5)	
From 6 000 to 16 000	2 (1.1)	5 (1.3)	7 (2.7)	14 (1.7)	
Mortality within last 12 months of family member					
No	173 (95.1)	318 (85.3)	220 (84.9)	711 (87.4)	<0.001
Yes	9 (4.9)	55 (14.7)	39 (15.1)	103 (12.6)	

^a^Min same as Minimum and Max same as maximum

**Table 2:** Socio-demographic, health, lifestyle and family history description of participants by ethnicity for 1 921 participants

Characteristic	Fulbe	Mbororo	General Population	Pooled	*p*-value
Health measures (x̄±SD)					
Height (cm)	163.3 (±8.7)	161.9 (±8.4)	162.6 (±8.2)	162.5 (±8.4)	0.01ª
Weight (kg)	55.0 (±14.1)	50.1 (±10.7)	58.6 (±12.9)	53.7 (±12.7)	<0.001ª
Waist Circumference (WC) (cm)	76.6 (±12.7)	74.4 (±10.5)	79.2 (±10.5)	76.3 (±11.2)	<0.001ª
Hip Circumference (HC) (cm)	91.7 (±11.2)	88.0 (±8.6)	92.1 (±10.3)	90.0 (±9.9)	<0.001ª
Waist-Hip-Ratio (WHR)	0.84 (±0.09)	0.85 (±0.10)	0.86 (±0.08)	0.85 (±0.09)	<0.001ª
Body Mass Index (BMI) (kg/m^2^)	20.5 (±4.6)	19.1 (±3.7)	22.1 (±4.3)	20.3 (±4.3)	<0.001ª
FCG Normal [FCG < 6.1 mmol/l (*n* = 1621)] (mmol/l)	4.9 (±0.6)	4.9 (±0.7)	5.0 (±0.6)	4.9 (±0.7)	0.001ª
BP (x̄±SD) (mmHg)					
Systolic Blood Pressure (SBP)	128.1 (±20.4)	127.9 (±23.8)	129.0 (±23.0)	128.3 (±22.8)	0.63ª
Diastolic Blood Pressure (DBP)	80.5 (±12.0)	80.4 (±14.3)	79.6 (±13.3)	80.2 (±13.5)	0.50ª
Gender, n (%)					
Male	139 (33.2)	286 (31.2)	212 (36.3)	637 (33.2)	
Female	280 (66.8)	632 (68.8)	372 (63.7)	1284 (66.8)	0.12^b^
Interview Language, n (%)					
French/English (official languages)	55 (13.1)	48 (5.2)	312 (53.4)	415 (21.6)	
Local language/Interpreter	364 (86.9)	870 (94.8)	272 (46.6)	1506 (78.4)	<0.001^b^
Age (x̄±SD)	35.7 (14.3)	36.0 (14.2)	36.4 (14.8)	36.4 (14.4)	0.79ª
Age group, n (%)					
20–39	274 (65.4)	596 (64.9)	376 (64.4)	1 246 (64.9)	
40–59	105 (25.0)	243 (26.5)	148 (25.3)	496 (25.8)	
60+	40 (9.6)	79 (8.6)	60 (10.3)	179 (9.3)	0.84^b^
Marital status, n (%)					
Single	45 (10.7)	54 (5.8)	81 (13.9)	180 (9.4)	
Married	329 (78.5)	768 (83.7)	431 (73.8)	1 528 (79.5)	
Separated	45 (10.7)	96 (10.5)	72 (12.3)	213 (11.1)	<0.001^b^
Level of education, n (%)					
Never	269 (64.2)	755 (82.2)	171 (29.3)	1195 (62.2)	
Attended school	150 (35.8)	163 (17.8)	413 (70.7)	726 (37.8)	<0.001^b^
Smoking habit, n (%)					
Abstainers	387 (92.4)	854 (93.0)	491 (84.1)	1732 (90.2)	
Former	23 (5.5)	52 (5.7)	55 (9.4)	130 (6.7)	
Current smokers	9 (2.1)	12 (1.3)	38 (6.5)	59 (3.1)	<0.001^b^
Alcohol drinking, n (%)					
Never	384 (91.7)	866 (94.3)	247 (42.3)	1497 (77.9)	
Occasional (drank in past 12 months) & daily drinkers	35 (8.3)	52 (5.7)	337 (57.7)	424 (22.1)	<0.001^b^
10 min physical activity at work, n (%)					
Vigorous	164 (39.1)	383 (41.7)	301 (51.5)	848 (44.1)	
Moderate	113 (27.0)	271 (29.5)	169 (28.9)	553 (28.8)	
Low	142 (33.9)	264 (28.8)	114 (19.5)	520 (27.1)	<0.001^b^
Walks for at least 10 min, n (%)					
No	163 (38.9)	316 (34.4)	121 (20.7)	600 (31.2)	
Yes	256 (61.1)	602 (65.6)	463 (79.3)	1 321 (68.8)	<0.001^b^
Vegetable intake/week, n (%)					
High (5–7 days/week)	260 (62.1)	470 (51.2)	244 (41.8)	974 (50.7)	
Moderate (3–4 days/week)	118 (28.2)	301 (32.8)	213 (36.5)	632 (32.9)	
Low (0–2 days/week)	41 (9.8)	147 (16.0)	127 (21.7)	315 (16.4)	<0.001^b^
Fruit intake/week, n (%)					
High (5–7 days/week)	104 (24.8)	146 (15.9)	127 (21.8)	377 (19.6)	
Moderate (3–4 days/week)	103 (24.6)	226 (24.6)	138 (23.6)	467 (24.3)	
Low (0–2 days/week)	212 (50.6)	546 (59.5)	319 (54.6)	1077 (56.1)	<0.001^b^
Always added salt at table, n (%)					
No	342 (81.6)	756 (82.4)	463 (79.3)	1561 (81.3)	
Yes	77 (18.4)	162 (17.6)	121 (20.7)	360 (18.7)	0.32^b^
Always added sugar to tea/coffee, n (%)					
No	337 (80.4)	766 (83.4)	434 (74.3)	1537 (80.0)	
Yes	82 (19.6)	152 (16.6)	150 (25.7)	384 (20.0)	<0.001^b^

ª*p*-value based on ANOVA

^b^ *p*-value based on chi square test

**Table 3:** Statistical significant variables of all pairwise differences between ethnic groups at Post Hoc testing

Parameter	Ethnic group	Contrast	Tukey *p*-value	Tukey 95% CI
Height, cm	Mbororo vs Fulbe	−1.395	0.01	−2.56 to −0.23
Weight, Kg	Mbororo vs Fulbe	−4.883	<0.001	−6.57 to −3.19
	General vs Fulbe	3.597	<0.001	1.76 to 5.43
	General vs Mbororo	8.480	<0.001	6.96 to 9.99
Waist Cir, cm	MbororovsFulbe	−2.149	<0.001	−3.67 to −0.62
	General vs Fulbe	2.614	<0.001	0.95 to 4.27
	General vs Mbororo	4.764	<0.001	3.39 to 6.13
Hip Cir, cm	Mbororo vsFulbe	−3.705	<0.001	−5.05 to −2.35
	General vsMbororo	4.132	<0.001	2.91 to 5.34
WHR	General vs Fulbes	0.026	<0.001	0.01 to 0.04
	General vs Mbororo	0.015	<0.001	<0.001 to 0.02
BMI, Kg/M^2^	Mbororo vsFulbe	−1.453	<0.001	−2.02 to −0.88
	General vs Fulbe	1.559	<0.001	0.94 to 2.17
	General vs Mbororo	3.013	<0.001	2.50 to 3.52
FCG, mol/l	General vsFulbe	0.117	0.02	<0.001 to 0.22
	General vs Mbororo	0.144	<0.001	0.05 to 0.23

**Table 4:** Age and gender standardised percentage prevalence of cardio metabolic risk factors by ethnicity and gender

Variable, n (%)	Fulbe, 419 (21.8)		Mbororo, 918 (47.8)		General population, 584 (30.4)		Pooled, 1 921 (100.0)	
	Men, 139	Women, 280	Men, 286	Women, 632	Men, 212	Women, 372	Men, 637	Women, 1284
Raised blood glucose								
IFG, 224 (7.8)	7.7	6.5	7.0	8.0	8.2	8.7	7.5	8.0
Diabetes,76 (3.1)	3.6	3.8	2.8	2.6	3.9	2.6	3.3	2.9
Raised blood pressure								
Pre-tensive, 694 (22.2)	28.3	21.9	25.6	18.3	26.0	22.2	26.4	20.1
Hypertensive, 592 (24.2)	26.2	25.2	24.5	24.8	24.9	23.0	25.0	24.3
BMI								
Underweight, 715 (24.3)	20.2	24.9	31.1	34.0	12.5	9.8	22.4	24.9
Overweight, 168 (6.1)	3.3	10.5	2.0	3.3	9.0	11.2	4.6	7.1
Obese, 59 (2.2)	2.3	4.0	0.6	1.0	2.4	4.5	1.5	2.6
SIR from WHR 725 (26.4)	19.3	29.1	19.9	26.5	19.0	36.1	19.7	30.0
IR from WC, 267 (9.5)	6.0	11.6	1.1	11.0	4.4	17.0	3.3	13.0
SIR from WC, 208 (7.6)	2.7	14.8	0.4	7.6	1.2	15.7	1.2	11.6

Statistical significance for F-test between groups (ANOVA, Bonferroni: Table 3) was observed for mean values of height, weight, WC, HC, WHR, BMI, and FCG and after post hoc tests between general population with Fulbe and general population with Mbororo for weight, WC, WHR, BMI, and normal FCG and for HC only between general population and Mbororo. Differences were also observed in participants of Peul descent (Fulbe & Mbororo) for height, weight, WC, HC, and BMI (Table 3).

### Consumption of alcohol

Occasional/daily alcohol consumption was often in the general population followed by the Fulbe (Table 2). The highest mean consumption (number of standard alcoholic bottles/week) was observed among the Fulbe (6.5, CI:2.9-10) followed by general population (4.7, CI:3.9-5.5) and Mbororo (4.1, CI:2.8-5.5). Men were often occasional/daily drinkers of alcohol than women; general population (62.3 and 55.1%; *p* = 0.09), and *p* < 0.001 for Fulbe (17.3 and 3.9%), Mbororo (11.2 and 3.3%), pooled data (29.5 and 18.5%) respectively. Alcohol consumption was pronounced in older (Mbororo and general population) and younger aged groups (Fulbe).

### Consumption of tobacco

Most participants were abstainers (Table 2). Proportion of current smokers was highest among the general population followed by Fulbe. Men were more often current smokers than women (Fulbe: 5.8% versus 0.4%, Mbororo: 3.9% vs 0.2%, general population: 15.6% vs 1.3% and pooled data: 8.2% vs 0.6%) with *p* < 0.001 for all the groups.

### Dietary habits

#### Intake of vegetables

We observed high vegetable intake of more than 50% for the Fulbe and Mbororo and 41.8% for general population (Table 2). No significant differences were observed in men and women (Fuble: 59 and 63.6%, *p* = 0.58, Mbororo: 52.5 and 50.6%, *p* = 0.87, general population: 38.7 and 66.4%, *p* = 0.48 and pooled data: 49.3 and 51.4% women, *p* = 0.59).

#### Intake of fruits

Low fruit intake (<50%) was observed in all the groups (Table 2) and highest fruit intake was among the Fulbe (24.8%). No significant differences were observed between men and women (Fulbe: 47.5 and 52.1%, *p* = 0.48, Mbororo: 57.3 and 60.4%, *p* = 0.54, general population: 51.4 and 56.5%, *p* = 0.49, with pooled data: 53.2 and 57.5%, *p* = 0.15 respectively).

#### Always added salt to food at table

Always added salt to food varied with men consumption less than for women (Table 2): Fulbe (16.6 and 19.3%, *p* = 0.49), Mbororo (17.5 and 17.7%, *p* = 0.93), pooled data (16.8 and 19.7%, *p* = 0.12) and general population (16 and 23.4%, *p* = 0.03) respectively.

#### Always added sugar to tea/coffee

Always added sugar to tea/coffee varied (Table 2) with men consumption higher than for women, Mbororo: 20.3 and 14.9%, *p* = 0.04, Fulbe: 22.3 and 18.2%, *p* = 0.32; general population: 26.4 and 25.3%, *p* = 0.76; pooled data: 22.8 and 18.6%, *p* = 0.03, respectively.

#### Physical activity at work for at least 10 min

Vigorous physical activity at work was practiced mostly by the general population, to a lesser extent by the Mbororo (Table 2) and often by men with significant differences (*p* < 0.001) except for the Fulbe (*p* = 0.18). Women were most often physically inactive when compared to men.

#### Walked for at least 10 min

Majority of participants in all the groups walked daily continuously for at least 10 min, with highest observed in the general population, and Mbororo (Table 2). Men practiced walking more than women; (Fulbe: 72.7 and 55.4%, *p* < 0.001, Mbororo: 78 and 60%, *p* < 0.001, general population: 84.4 and 76.3%, *p* = 0.02 respectively).

#### Prevalence of IFG and diabetes

Prevalence of Impaired Fasting Glucose (IFG) and diabetes in this rural population was respectively: 7.8 and 3.1%, Fulbe: 6.9 and 4.0% (highest), Mbororo: 7.7 and 2.6%, and general population: 8.5% (highest) and 3.1%. Higher rates were often among men than women, except for Fulbe (Table 4) with no significant difference (Fulbe, *p* = 0.77, Mbororo, *p* = 0.35, general population, *p* = 0.17 and pooled data, *p* = 0.23). Prevalence of diabetes peaked at 40–59 years for all groups. Prevalence of undiagnosed diabetes was 79% in the study population, 78.3% among Fulbe, 74.2% among Mbororo and 86.4% in the general population; peaked at 20–39 years age group for Fulbe (81.8%) and Mbororo (84.6%) and 40–59 years age interval for general population (88.9%).

#### Prevalence of hypertension

Little variation in mean SBP and DBP was observed in the three groups (Table 2). Men had higher SBP than women, Fulbe (131.8 mmHg and 126.2 mmHg), Mbororo (133.4 mmHg and 125.4 mmHg), general population (132.1 mmHg and 127.3 mmHg) and for pooled data (132.1 mmHg and 127.3 mmHg). High blood pressure and pre-hypertension varied from one group to another, with the Fulbe 23.9 and 25.3% (highest), Mbororo 20.5 and 24.2% and general population 23.4 and 23.4%. Pre-hypertension was higher than hypertension in all groups (Table 4). Men were often hypertensive and pre-hypertensive than women in all groups (Fulbe, Mbororo and pooled data: *p* < 0.001, general population, *p* < 0.001 respectively). High blood pressure increased with increased in age. Undiagnosed hypertension peaked at 20–39 year interval for all the groups and was 97.1% in the study population; 96.3% for the Fulbe, 98.2% for Mbororo and 96% for general population.

#### Prevalence of overweight/obesity, IR/SIR from WC or WHR

Highest mean BMI was observed among the general population followed by Fulbe (Table 2). Peul men had higher mean BMI values than women in contrast to general population (Fulbe; 20.6 and 20.5%, *p* = 0.68, Mbororo; 19.3 and 18.9%, *p* = 0.21; general population; 21.7 and 22.3%, *p* = 0.07). Peul men had higher mean WC values than women; Fulbe 78% versus 75.8%, *p* = 0.09; Mbororo 76.0% against 73.7%, *p* < 0.001 in contrast to general population 78.1% compared to 79.8%, *p* = 0.05. Men had smaller HC mean values than women in all groups; Fulbe: 91.3% against 91.9%, *p* = 0.62, Mbororo: 87.5% versus 88.2%, *p* = 0.29 and general population 90.4% compared to 93.1%, *p* < 0.001. Mean WHR values for men were higher than for women in all groups; Fulbe: 0.85 and 0.82, *p* < 0.001, Mbororo: 0.87 and 0.84, *p* < 0.001, general population: 0.87 and 0.86, *p* = 0.33.

Overweight and obesity was respectively: pooled data 6.1 and 2.2%, Fulbe 7.0 and 3.4%, Mbororo 2.9 and 0.7%, general population 10.3 and 3.7%. Overweight/obesity was more pronounced in women than men for all the ethnic groups (Fulbe: *p* < 0.001, Mbororo: *p* = 0.09, general population: *p* = 0.28 and pooled data: *p* < 0.001). Increased Risk (IR) and Substantially Increased Risk (SIR) from WC was: pooled data 9.5 and 7.6%, Fulbe 10 and 10.1%, Mbororo 7.5 and 5%, and general population 12.1 and 9.8%. More often women had SIR from WC than men, *p* < 0.001 for all groups. SIR from WHR was: pooled data 26.4%, Fulbe 25.8%, Mbororo 24.5% and general population 29.5%. Women more often had SIR from WHR than men, general population (*p* < 0.001), Fulbe (*p* = 0.03) and Mbororo (*p* < 0.001) and pooled data (*p* < 0.001). The Mbororo were the least obese and overweight and often underweight (Table 4). Women were often underweight than men among the Fulani but men were often underweight than women for the general population (Table 4). Underweight decreased with age while overweight/obesity increased for the Fulbe. Underweight peaked at ≤60 years followed by 20–39 age bracket and overweight/obesity peaked at 40–59 years for the Mbororo. Underweight increased with age and overweight/obesity peaked at 40–59 years for the general population.

## Discussion

There is a paucity of knowledge on the risk factors of NCDs in indigenous populations of Sub Saharan Africa. This study provided population based comparative data on risk factors of NCDs for indigenous Mbororo and non-autochthonous populations in rural Cameroon.

Statistical significance for F-test between groups (ANOVA, Bonferroni) and post hoc tests suggested ethnic variations; implying the importance of ethnicity in predicting these variables for this population.

The prevalence of diabetes amongst the study population was lower than the prevalence among Australia Aboriginal and Torres Strait Islander peoples (≤30%) [36]. Also, it was lower than global prevalence (9%), Cameroon prevalence (6.3%) in 2014 [37] and prevalence of 4.9% in 2013 for 20–79 year-olds [38]. The prevalence among the settled Fulani (Fulbe) was higher than for the nomadic Fulani (Mbororo). This may be due to resultant effect of lifestyle change from nomadic to settle life and the inadvertent risks involved in such transition without accompanying measures. Gender differences in prevalence were observed in all ethnic groups and this in-line with report from systematic reviews [39]. The standardised prevalence was lower than the projected (4.8%) for Cameroon by 2030 [40]. Given the high undiagnosed rates, and the high prevalence of IFG and diabetes especially among the younger population, the diabetes epidemic may be unfolding and increasing in rural milieu. Raised blood glucose is a risk factor for NCDs and diabetes is a risk factor for CVDs with complications when not diagnosed early [1, 2, 5]. The consequences are increase in hospitalisations, morbidity and mortality especially in indigenous rural people with resultant shorter life expectancies.

Mean SBP for men was higher than 131.3 mmHg for men and for women it was lower than 127.3 mmHg for Cameroon population in 2008 [41]. Prevalence of SBP increased in men since 2008 but stagnated in women. Prevalence of hypertension in all groups was lower than 2010 estimates of 39.6% in men and 37.2% women [42]. Age standardised prevalence in 2003 for rural populations of Cameroon were 34.1 and 44% [43] suggesting a reduction of rural prevalence in a ten year period of 9.8 and 19.0% in women and men respectively, probably due to methodological differences. Our standardised rural estimates were higher than WHO global status report on NCDs of 21.6% for hypertension in Cameroon [37] and standardised prevalence of 20.4% in urban Cameroonian population in 2007 [23]. Hypertension awareness varied (1.8 to 4%) though lower than the 7 to 56% (pooled 27%) from studies in Sub Saharan Africa between 2000 and 2013 [44]. Raised blood pressure is a risk factor for NCDs and CVDs. Hypertensive individuals compared to normotensive individuals have a twofold, fourfold, and sevenfold increased risk of developing coronary artery disease, congestive heart failure and cerebrovascular and stroke respectively [45]. The high prevalence of raised blood pressure, undiagnosed cases and clustering in the age group of 20–39 years indicates long term complications, disability, morbidity and mortality.

Mean BMI for all groups was lower than estimates from global statistics of NCDs, 23.8 kg/m^2^ for men and 25.1 kg/m^2^ for women and pooled 24.4 kg/m^2^ for Cameroon [37]. The BMI for this population is within the recommended interval of 21–23 kg/m^2^ for populations striving for optimal health and individual goals of 18–24.9 kg/m^2^. Higher mean BMI values for men as compared to women coupled with higher prevalence of underweight among Peul especially women probably indicate poor nutrition, poor health and susceptibility to infectious diseases in crowded households, more illnesses and lower life expectancies. This may explain high mortality for Mbororo households in this study. Highest prevalence of overweight observed in the general population was lower than WHO 2014 estimates of 22.1% for men and 36.9% for women and pooled data 25.1% [37]. Fulbe women were twice as overweight as men. Cultural beliefs restrict Fulbe and Mbororo women from physically exacting tasks. Many lead a more sedentary lifestyle as compared to their men folks which results in weight gain and increased health risk. This was corroborated by studies in Fulani population in Nigeria [46]. Prevalence of obesity defined by BMI was lower than the obesity estimated from global statistics of NCDs, 4.9% for men to 14.3% in women [37]. Cut-off points from BMI indicate that obesity is rare among the Fulbe and Mbororo. Central obesity as defined by WHR was not rare among Fulbe and Mbororo. In clinical settings BMI is widely used to ascertain degree of weight increased associated with risk of cardiovascular complications. Current BMI cut-off points maybe inappropriate for the Fulani. Validated population studies to define ethnic specific cut-off points for this population are warranted to identify which of WHR or WC maybe appropriate for Fulbe and Mbororo in clinical settings. Overweight/obesity is driver of NCDs and cardiovascular diseases. The low prevalence in the Fulani population may imply that they are less affected by these diseases. Nonetheless, these diseases are also determined by other factors which may not favour the Fulani population like the environment, genetic pre-disposition, early life experiences and life course factors [21].

Tobacco consumption was lower than 2012 prevalence of 16.6% for adults aged 15 years and over [37]. It may be higher in the report because of inclusion of the 15–19 age interval not captured in this study. Majority participants (90.2%) were non-smokers suggesting a higher consumption of tobacco in urban areas (24.6% in 2007) [23] than rural areas (9.8% in 2013) in Cameroon. Current smoking increased with age and peaked at 40–49 years for Fulbe and Mbororo, and ≤60 years for general population (highest rates) though still lower than for urban centres where older groups reported less current smoking [23]. The majority of smokers were often men in all groups. Smoking starts at tender age is addictive and a lifestyle risk factor for NCDs and CVDs. Increase in smoking rates may be foreseen among the Fulani though their traditional beliefs and religious practices forbid smoking but as they move to urban centres and adopt western lifestyles coupled with dislocation and loss of land and cattle, smoking rates may increase further compounding the epidemiology of NCDs in this population.

Considerable difference was observed in alcohol consumption between this rural population and urban population of Cameroon (65.1%) in 2007 [23]. Global statistics on NCDs estimated heavy 13.3% episodic alcohol consumption in 2010 for Cameroonians aged ≤15 years among men, 3.5% among women and 8.4% for both sexes whilst for our study population it was 29.5% among men and 18.5% among women and 22.1% in pooled data. This may be accounted for by differences in methodology and target populations. Alcohol consumption is increasing among the aboriginal societies as observed with the San in Botswana and Namibia probably as a result of poverty accentuated by loss of land and livelihoods with no workable alternatives and exposure to non-indigenous lifestyle [1]. Alcohol consumption has not been documented among the Fulani. Their traditional and religious practices abhor alcohol consumption but with transitional lifestyle they are indulging in alcohol consumption with attendant increase rates of NCDs and related complications in later life.

Vegetable intake was higher than fruit intake in all groups. Older people tended to report more vegetables consumption than younger people. Salt and sugar intake was pronounced among the younger age groups. Sugar was often taken with tea/coffee mostly drank daily by Fulbe and Mbororo at breakfast and all daylong as a cultural habit. Most often salt intake was with vegetables and roasted beef, a cultural trait of Fulbe and Mbororo. The high consumption of salt and sugar, and low intake of fruits especially among the young may translate into more cases of hypertension, diabetes, CVDs and NCDs complications in later life.

Rates of vigorous physical activity at work were higher than the levels of physical activity estimated by global statistics on NCDs in 2010 for adult Cameroonians aged 18 years and above, 20.9% men, 37.7% women and 29.3% both sexes. This population also reported high continuous daily walks of at least ten minutes. Their main activity was subsistent farming (≤30%). Endemic underweight maybe accounted for by poor nutrition, high levels of vigorous physical activity and daily walking with a net expense in energy than intake. Low levels of physical activity may increase among the Fulbe and Mbororo with the rapid transition from nomadic to settled life and change of lifestyle and nutritional habits with increasing access to processed foods. This will translate into overweight/obesity responsible for increasing rates of NCDs and CVDs.

The lifestyle of Mbororo/Fulbe is undergoing a profound transition in terms of social mobility, feeding habits and health. Studies suggest differences in chronic medical conditions, age and sex differences in associations may be explained by demographic and socioeconomic factors [47, 48]. The higher prevalence of NCD risk factors in settled Fulani (Fulbe) when compared to the nomadic Fulani (Mbororo) may indicate a greater susceptibility to NCDs with settled life than the general population due to change of lifestyle, genetic predisposition or factors linked to early life development.

## Conclusion

This study points out the preponderance of ethnicity, enculturation and exposure as determinants in the epidemiology of NCDs and provided evidence-informed data on distribution and patterning of NCD risk factors in rural Aboriginal populations. The prevalence of NCD risk factors differ from previous studies. It clustered in the age groups of 40–59 to 60 > years and amongst men except for IR/SIR from WC and SIR from WHR that clustered amongst women. Differences exist in the distribution of risk factors of NCDs between indigenous and non-indigenous populations. A changed from nomadic (Mbororo) to settled (Fulbe) life of Fulani has possibly resulted in increased prevalence of seven NCD risk factors (raised blood glucose, raised blood pressure, low levels of habitual physical activity, occasional and daily alcohol consumption, current tobacco smoking, overweight/obesity, and diet – always added sugar to tea/coffee, always added salt at table, high levels of vegetable/fruit intake). These risk factors may even increase further and at a faster rate in Mbororo than in the Fulbe due to rapid transition from nomadic to settled life, lack of education and health care.

The population of Africa especially Sub Saharan Africa is rapidly increasing and more people will be in urban areas, adapt a western lifestyle and develop NCDs. Given the uncertainty in disease epidemiology, rural indigenous people cannot deal with the burden of NCDs. Furthermore, NCDs risk factors overlap with other causes of ill health such as infectious diseases. To our knowledge this is the first comparative study examining the distribution of NCDs risk factors in indigenous Mbororo and other populations. More evidenced informed data needed for appropriate ethnic specific interventions to turn the tide against the silent and bourgeoning pandemic of NCDS.

Strengths of the study are many. The study is a quantitative population based study with a response rate of 90.8% focusing on an indigenous population hitherto not considered as a separate and distinct group. The high response rate was probably due to community sensitization prior to study through traditional authorities and religious leaders’ hegemony. Cardio-metabolic values were standardised permitting comparability. It also allowed for comparison with the non-indigenous population living in the same geographic location and almost at same epidemiologic transition phase. Distribution of risk factors among Fulani participants further divided into Fulbe and Mbororo and older adults have also been considered, for the most part neglected in NCD analysis. The WHO STEPwise approach permitted comparison with other similar studies. Standardised WHO questionnaires and international guidelines were used for the definition of diabetes, hypertension, and weight measures. The study gives insights into the prevalence of NCD risk factors in populations whose traditional health system is unknown because of their discrete, secretive, migratory and indigenous lifestyle and provided baseline data in tackling the NCD epidemic. Trained enumerators conversant with the widely spoken local Fulfulde language administered the questionnaires.

This is a cross-sectional study and limited in examining multiple scale causal mechanisms. Change in cut-off point for definition of diabetes from 6.1 to 7.0 mmol/l by WHO/IDF in 2010 makes generalizability with previous studies problematic. Instant translation of questionnaires from French into local language might have led to poor understanding and introduction of a bias. Though we conducted at least two measurements and took the average our instrument standardisation method for weight, height, blood pressure, WC and HC and were suited for field study and might have introduced non-differential bias. In clinical practice, diagnosis of diabetes necessitates laboratory methods or use of A1C measurements which gives mean glucose levels for two to three months or multiple measurements on different occasions we took two measurements at interval of two days at least. We are aware of quantification of fruit and vegetable consumption in days of intake per week and not servings or grams which would have been confusing to participants who do not measure food consumed in this manner. Some data was collected through respondent self-reported lifestyle factors prone to recall bias. Fulani holistic beliefs consider wealth as land, cattle, etc. which was difficult to evaluate. Road accessibility may introduce a bias but comparison with other non-autochthonous populations resident with the indigenous people was taken into consideration.

## Abbreviations

ANOVA: Analysis of variance
BMI: Body mass index
CI: Confidence interval
CVDs: Cardiovascular diseases
DBP: Diastolic blood pressure
FCG: Fasting capillary glucose
HC: Hip circumference
IDF: International Diabetes Federation
IFG: Impaired fasting glucose
IR: Increased risk
NCDs: Non communicable diseases
PPS: Probability proportional to size
SBP: Systolic blood pressure
SD: Standard deviation
SIR: Substantially increased risk
UN: United Nations
WC: Waist circumference
WHO: World Health Organisation
WHR: Waist-hip ratio
